# Chitosan Nanoparticles for Topical Drug Delivery in Chemotherapy-Induced Alopecia: A Comparative Study of Five Repurposed Pharmacological Agents

**DOI:** 10.3390/ph18071071

**Published:** 2025-07-21

**Authors:** Salma A. Fereig, John Youshia, Ghada M. El-Zaafarany, Mona G. Arafa, Mona M. A. Abdel-Mottaleb

**Affiliations:** 1 Department of Pharmaceutics and Pharmaceutical Technology, Faculty of Pharmacy, The British University in Egypt (BUE), El Sherouk City 11837, Egypt; salma.fereig@bue.edu.eg (S.A.F.);; 2 Department of Pharmaceutics and Industrial Pharmacy, Faculty of Pharmacy, Ain Shams University, Cairo 11566, Egypt; 3 Nanotechnology Research Center (NTRC), The British University in Egypt, El Sherouk City 11837, Egypt; 4 Chemotherapeutic Unit, Mansoura University Hospitals, Mansoura 35516, Egypt; 5 EFS, INSERM, UMR 1098 RIGHT, Université de Franche-Comté, F-25000 Besançon, France

**Keywords:** chemotherapy-induced alopecia, anagen effluvium, chitosan nanoparticles, drug delivery, phenobarbital

## Abstract

**Background/Objectives**: Chemotherapy-induced alopecia is a common and distressing side effect of cancer treatment, significantly impacting patients’ psychological well-being. Nanocarriers offer a promising strategy for targeted drug delivery to hair follicles, while chitosan nanoparticles have demonstrated hair-growth-promoting properties. This study explores the potential of chitosan nanoparticles as a topical delivery system for five pharmacological agents—phenobarbital, pioglitazone, rifampicin, N-acetylcysteine, and tacrolimus—to prevent chemotherapy-induced alopecia. **Methods**: Drug-loaded chitosan nanoparticles were prepared using the ionic gelation technique and characterized by particle size, zeta potential, entrapment efficiency, FT-IR spectroscopy, and TEM imaging. Their efficacy was assessed in a cyclophosphamide-induced alopecia model in C57BL/6 mice through macroscopic observation, histopathological examination, and scanning electron microscopy of regrown hair. **Results**: The prepared particles were spherical, cationic, and between 205 and 536 nm in size. The entrapment efficiencies ranged from 8% to 63%. All five drugs mitigated follicular dystrophy, shifting the hair follicle response from dystrophic catagen to dystrophic anagen. Phenobarbital demonstrated the most significant hair regrowth and quality improvements, followed by N-acetyl cysteine and pioglitazone. Tacrolimus showed moderate efficacy, while rifampicin was the least effective. **Conclusions**: These findings suggest that phenobarbital-loaded chitosan nanoparticles represent a promising approach for the prevention and treatment of chemotherapy-induced alopecia, warranting further investigation for clinical applications.

## 1. Introduction

Over the past century, advancements in oncology research have significantly increased cancer survival rates worldwide [1]. However, chemotherapy-induced adverse effects remain a major challenge, with chemotherapy-induced alopecia (CIA) being one of the most distressing and stigmatizing side effects. Despite extensive research, no universally effective preventive protocol for CIA has been established [2]. The only FDA-approved protocol for CIA is scalp cooling. This technique works by vasoconstriction within the scalp, which minimizes the delivery of cytotoxic agents to hair follicles (HFs); hence, it can provide follicular protection. However, most cancer patients still choose not to use scalp cooling because of its inefficacy, especially with patients who receive combination therapy, and side effects that include headache, dizziness, local pain, skin rash, and heavy-headedness [2,3]. Clinical trials have revealed that most patients who have undergone scalp cooling still need to wear wigs during and after chemotherapy administration [3,4,5,6,7]. Since the 1990s, researchers have explored various pharmacological approaches to mitigate CIA, including monoclonal antibodies [8], N-acetyl cysteine (NAC) [9,10], cyclosporine [11], AS101 [12], minoxidil [13], fibroblast growth factors [14], geldanamycin [15], bimatoprost [16], epinephrine [17], and cellium [18], as a preventive pre-chemotherapy protocol for alopecia. Unfortunately, most clinical trials have reported limited success in CIA prevention [19,20,21]. Owing to the different underlying pathology of CIA compared to other types of alopecia, the conventional management of alopecia using minoxidil or finasteride is not suitable for CIA. Minoxidil was proven to enhance hair regrowth after HF recovery; however, it has no role in preventing CIA or accelerating follicular recovery via alteration of the dystrophic pathway that resulted from the cytotoxic insult [22,23,24]. Finasteride is a 5-alpha reductase inhibitor; thus, it plays a role in hormonal changes within the body. Therefore, it would be too risky to use it for CIA management, as it could enhance cancer progression, especially in hormone-sensitive cancer types [25].

Nanocarriers refer to drug delivery vehicles within the nanometer range and offer an advantage against CIA, as they can be tailored to target a specific site—the HFs in our case—with minimal drug accumulation in non-target areas [26]. Drug delivery researchers have proven that certain types of nanocarriers, such as polymeric nanospheres and nanocapsules, solid–lipid nanoparticles, and nanostructured lipid carriers, are more suitable for HF targeting than others, such as nanoemulsions, liposomes, and nanocrystals [27]. Polymeric nanoparticles formulated from chitosan (CS) are of particular interest, as chitosan has been reported to have hair-growth-promoting activity [28] due to its tissue-regenerative properties [29,30]. This was confirmed in a previous project conducted by our group, where we used TAC-loaded chitosan nanoparticles (CS NPs) for the topical treatment of psoriasis [31,32,33]. We noticed an interesting observation: significant hair growth in mice. This effect was attributed to both CS and TAC. Also, CS NPs were previously reported to have a potential for follicular targeting while loaded with minoxidil (6-fold more than the control solution) [34]. Therefore, CS NPs represent a promising system for the synthesis of a protective or therapeutic formulation for CIA. Based on our literature review, several pharmacological agents might be promising in CIA prevention or treatment.

Phenobarbital (PHB) is a drug that belongs to the class of barbiturates, used mainly as anti-epileptics and for the treatment of insomnia and alcohol withdrawal [35]. Pioglitazone (PGZ) is an oral hypoglycemic drug that belongs to the class of thiazolidinediones, used for the management of type 2 diabetes [36,37]. Rifampicin (RIF) is an antibiotic used for the treatment of leishmaniasis and *Mycobacterium tuberculosis* [38,39]. All three agents (PHB, PGZ, and RIF) are agonists to different receptors (constitutive androstane receptor (CAR), peroxisome proliferation receptor gamma (PPARγ), and pregnane X receptor (PXR), respectively) that belong to the ATP-binding cassette (ABC) receptor family. [40,41,42]. Upregulating the ABC receptors is associated with the efflux of chemotherapeutic drugs from HFs, hence limiting the chemotherapeutic insult to HFs [43,44,45]. It was even recommended to exploit HF-targeting formulations synthesized by nanotechnology to achieve the desired effects [46].

NAC is a derivative of the amino acid L-cysteine [47]. It is used as a mucolytic, liver and kidney support agent, and an antidote for paracetamol liver toxicity [9]. Previous research demonstrated that NAC (administered either topically or injected S.C.) provided protective effects against CIA induced by cyclophosphamide (CYP), but not by cytarabine (ARA-c), in a rat model [9]. Also, oral NAC was reported to have a protective effect against doxorubicin-induced cardiotoxicity, mutagenicity, and alopecia [48]. NAC was formerly used as an antidote against paracetamol, doxorubicin, CYP, and even irradiation; thus, it is described as a cell protectant from cytotoxicity. Despite its potential, further investigations into NAC’s mechanism and efficacy in CIA prevention are lacking.

Tacrolimus (TAC) is an immunosuppressant macrolide, usually used to prevent post-transplant and post-graft rejection [49,50]. It is also used topically for the treatment of atopic dermatitis and eczema, as well as psoriasis in an off-label scope [51,52,53,54]. Being an immunosuppressant, TAC is extensively reported as a potential medication for the management of alopecia areata, which has an autoimmune underlying etiology, yet with limited effectiveness [55,56]. TAC has been suggested for the treatment of alopecia in several studies [31,32,33]. However, the potential of TAC to enhance hair growth was previously described to be via vasoconstriction [57]. This could be beneficial in minimizing the amount of cytotoxic agents that invade the HF cells. Moreover, TAC was reported to induce anagen and protect against CIA in animal experiments [58]. The exact mechanism is not described yet, and to the best of our knowledge, no further studies have been conducted on CIA using TAC.

Despite ongoing research efforts, a significant gap remains in the effective management of CIA. Most of the existing studies are truncated, and further exploration is needed to develop a promising formulation for the management of CIA. Owing to the unique pathophysiological changes that happen with HF cells after chemotherapeutic administration, which are totally different from other types of alopecia, we think that there is a strong rationale to explore new approaches in CIA management, whether for CIA prevention or for the acceleration of follicular recovery.

In this context, we hypothesized that one or more of the selected agents (PHB, PGZ, RIG, NAC, and TAC) could mitigate the cytotoxic damage to HF cells during the chemotherapeutics regimen period, whether by drug efflux, antioxidant protection, or immunomodulation. The suggested agents are thought to be more promising in comparison to other tested drugs in CIA prevention, such as CDK inhibitors, P53 inhibitors, 1,25-dihydroxyvitamin D3, minoxidil, and cyclosporine A, which have either proven ineffective or are limited to CIA induced by certain chemotherapeutic agents [11,13,14,23,59].

The incorporation of each agent into CS NPs may enhance the therapeutic outcome because of the tissue-regenerative properties of the CS itself, potentially facilitating HF recovery post-chemotherapy. Additionally, CS offers an ideal carrier for topical formulations that aim for local skin deposition within the skin layers and HFs, rather than transdermal absorption. Minimizing systemic absorption is crucial to prevent hindrance of the chemotherapeutic effect in cancer treatment.

To test our hypothesis, we constructed our study design to formulate CS NPs loaded with each of the selected active agents: PHB, PGZ, RIF, NAC, and TAC [9,46,60,61]. The prepared formulae were characterized in terms of particle size (PS), zeta potential (ZP), entrapment efficiency (EE%), drug loading (DL%), Fourier-transform infrared (FT-IR) analysis, and transmission electron microscope (TEM) examination. Their efficiency in the prevention and/or treatment of CIA or CYP-induced alopecia in the C57BL/6 mouse model was compared. The in vivo efficacy was assessed via visual observation of the progress of hair growth on the mice’s backs and the scoring of alopecia at different time points of the study. Moreover, we examined the quality of growing hair under a scanning electron microscope and assessed HFs and hair shaft quality by histopathological examination of skin samples at different time points.

This work aimed to develop a nanotherapeutic formulation to be applied topically before a chemotherapeutic regimen to prevent alopecia and be used afterwards as a treatment to promote hair regrowth. By improving the psychological well-being of cancer patients during treatment, this strategy may contribute to better overall treatment outcomes and disease prognosis.

## 2. Results

### 2.1. Characterization of Drug-Loaded Chitosan Nanoparticles

**Figure 1:**
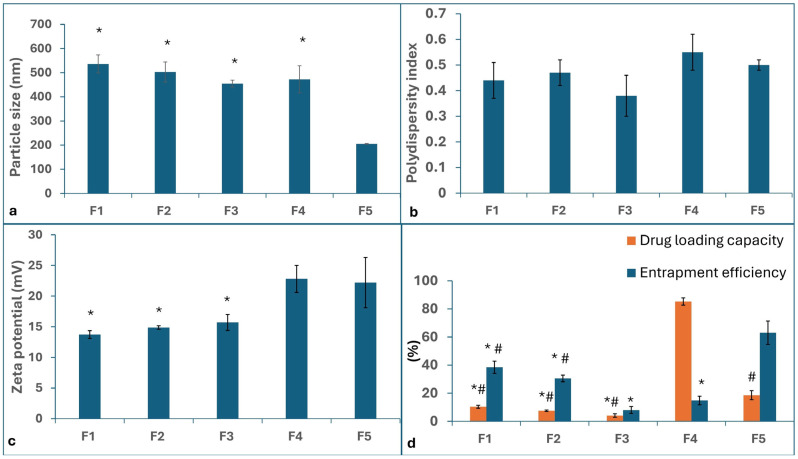
Characterization of drug-loaded chitosan nanoparticles; F1–F5 are loaded with phenobarbital, pioglitazone, rifampicin, N-acetyl cysteine, and tacrolimus, respectively: (**a**) particle size determined as intensity-weighted average, (**b**) polydispersity index, (**c**) zeta potential, and (**d**) entrapment efficiency and drug loading capacity. * Statistically significantly different from F5 (*p* ≤ 0.05). # Statistically significantly different from F4 (*p* ≤ 0.05).

CS NPs were successfully prepared using the modified ionic gelation technique. The PS, PDI, ZP, EE%, and DL% results are shown in Figure 1.

#### 2.1.1. Particle Size (PS), Polydispersity Index (PDI), and Zeta Potential (ZP)

The PS of all formulations ranged from 205 to 536 nm, while the PDI ranged from 0.38 to 0.55, which shows moderate-to-high polydispersity. One-way ANOVA showed a statistical difference among the different formulations (*p* ≤ 0.05). Tukey’s multiple comparisons test showed that F5 produced significantly smaller nanoparticles than the other formulations (*p* ≤ 0.05). No significant difference was found in PS among F1–F4.

All formulations showed a positive surface charge. The ZP ranged from 13.73 mV to 22.8 mV. One-way ANOVA showed that the results were significantly different (*p* ≤ 0.05). Tukey’s multiple comparisons test showed no statistically significant differences among F1–F3 (*p* > 0.05) and F4–F5 (*p* > 0.05), whereas F1, F2, and F3 were all statistically significantly lower than F4 and F5 (*p* < 0.05).

#### 2.1.2. Entrapment Efficiency (EE%) and Drug Loading (DL%)

The EE% of the formulations ranged from 8 to 63.07%. They were statistically significantly different from each other (*p* ≤ 0.05). This can be attributed to the nature of each drug and its solubility in the used co-solvent (propylene glycol) and in the acidic media of the formulation (pH = 3). The post hoc test showed that F1 was statistically non-significantly different from F2 (*p* > 0.05). Additionally, F3 was non-significantly different from F4 (*p* > 0.05), whereas all other multiple comparisons were significantly different from each other (*p* ≤ 0.05). The DL% results ranged from 4.08 to 85.23% and showed that F4 had a significantly higher DL% than all of the other formulations. F5 was second, showing a significantly higher DL% than F1–F3. F1’s DL% was significantly higher than that of F3, and no significant difference was found between F1 and F2, or between F2 and F3. The detailed chromatographic results used in the EE% calculations are provided in Table A1.

#### 2.1.3. Morphological Examination Using Transmission Electron Microscopy (TEM)

**Figure 2:**
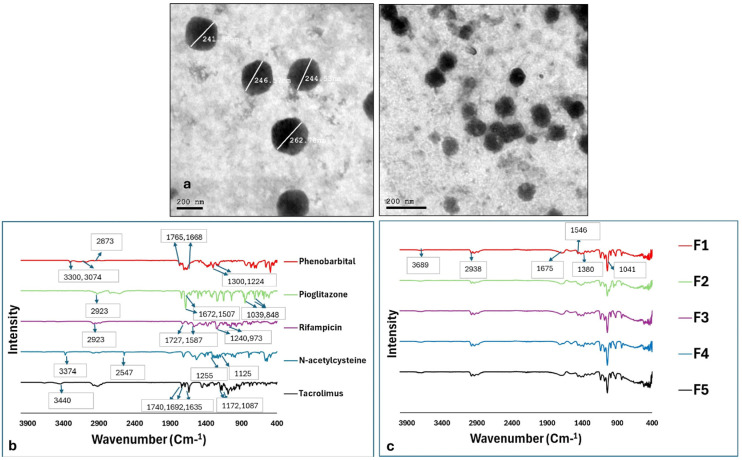
Physicochemical characterization of the prepared chitosan nanoparticles: (**a**) transmission electron microscope images of model chitosan nanoparticles; (**b**) Fourier-transform infrared spectra of individual drugs (phenobarbital, pioglitazone, rifampicin, N-acetylcysteine, and tacrolimus); (**c**) Fourier-transform infrared spectra of drug-loaded chitosan nanoparticles, where F1–F5 are loaded with phenobarbital, pioglitazone, rifampicin, N-acetylcysteine, and tacrolimus, respectively.

To observe the nanoparticles and verify their formation, microscopic analysis was conducted. TEM imaging revealed the presence of spherical nanoparticles with sizes ranging from 241 to 262 nm, in alignment with data determined by dynamic light scattering (Table 1). The TEM micrographs obtained from the model CS NPs are displayed in Figure 2a.

### 2.2. Fourier-Transform Infrared (FT-IR) Analysis

To evaluate the spectral characteristics of different functional groups, bond types, and band intensities, FT-IR analysis was conducted on the individual drugs (Figure 2b) and drug-loaded nanoparticles (Figure 2c). The FT-IR spectrum of PHB exhibited distinct bands at 3300 and 3074 cm^−1^ corresponding to symmetric and asymmetric N–H stretching, respectively. A low-frequency band at 1765 cm^−1^ was attributed to C=O stretching, while C–N bonds were identified at 1298 and 1224 cm^−1^ [62]. PGZ displayed characteristic stretching vibrations at 2923, 1507, 1672, and 1039 cm^−1^, associated with C–H, C–C, C–O, and C–N bonds, respectively. Additionally, a prominent bending vibration at 848 cm^−1^ corresponded to the =C–H bond 949494 [63]. The FT-IR spectrum of RIF revealed a C–H stretching band at 2923 cm^−1^ and an acetyl C=O stretching band at 1727 cm^−1^. Bands at 1587, 1376, and 1240 cm^−1^ were assigned to C=C, C–N, and C–O bonds, respectively. Furthermore, a bending absorption band at 973 cm^−1^ indicated the presence of a C=C double bond 959595 [64]. For NAC, a characteristic S–H stretching vibration appeared at 2547 cm^−1^, alongside N–H, C=O, and C–O bands at 3374, 1255, and 1125 cm^−1^, respectively (96,9796, 9796,97) [65,66]. The FT-IR spectrum of TAC exhibited –H stretching at 3440 cm^1^. C–O–C stretching was observed at 1172 and 1087 cm^−1^, with distinct C=O and C=C bands at 1740 and 1635 cm^−1^, respectively [67].

Regarding drug-loaded CS NPs, all of the spectra were similar, displaying the characteristic peaks of CS, including O–H and N–H stretching at 3689 cm^−1^, as well as C–H, C=O, C–O, and P–O stretching at 2938, 1675, 1380, and 1035 cm^−1^, respectively. Moreover, N–H and C–O bending vibrations were prominent at 1565 and 1409 cm^−1^ [68]. Notably, the characteristic peaks of the individual drugs disappeared in their respective drug-loaded CS NP spectra, indicating successful encapsulation.

### 2.3. In Vivo Comparison of the Efficacy of Chitosan Nanoparticles Loaded with Different Agents in the Prevention and/or Treatment of Chemotherapy-Induced Alopecia in an Animal Model

#### 2.3.1. Induction of Anagen VI to Induce Chemotherapy-Induced Alopecia

**Figure A1:**
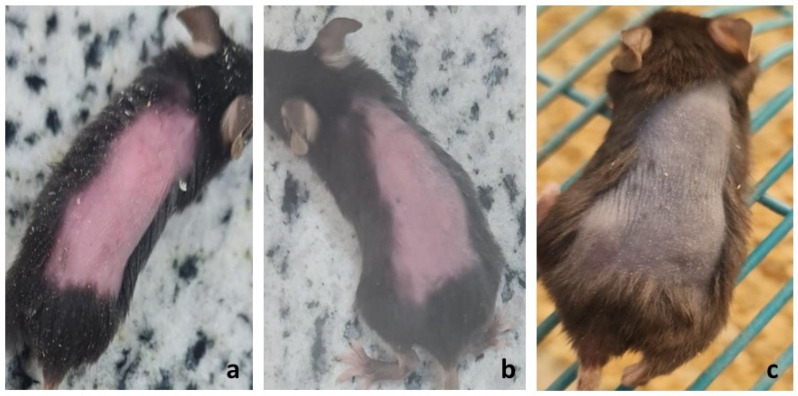
A representative mouse at different time points post-depilation during induction of chemotherapy-induced alopecia to specify the different stages of the hair cycle by observing the changes in skin color. (**a**) On day 0, with a pink skin color that indicates the induction of the anagen I phase. (**b**) On day 5, with a grey skin color that indicates the anagen IV phase. (**c**) On day 9, with a grey/black skin color that indicates the anagen VI phase.

The depilation process of adolescent mice with fully grown hair in the telogen phase (resting phase) induced a new hair cycle by the induction of anagen I. This was confirmed by the pink color of the skin after depilation (Figure A1a). By day 5, the mouse hair was transformed to the anagen IV phase, characterized by grey skin color (Figure A1b). By day 9, the mouse hair reached the anagen VI phase, characterized by dark grey or black skin color (Figure A1c). On day 9, all of the treatment groups and the negative control group were injected with CYP to undergo either the dystrophic catagen or the dystrophic anagen pathway [69,70].

#### 2.3.2. Visual Assessment of Hair Loss and Regrowth Pattern

**Figure 3:**
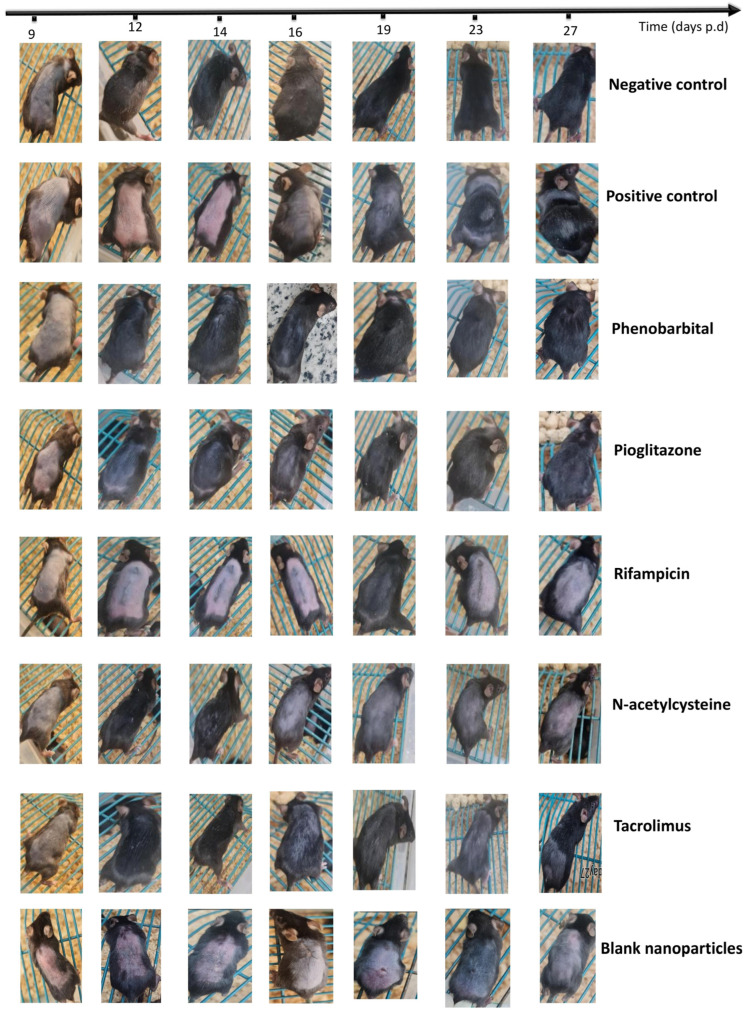
Representative mice from each group at different time points post-depilation: the negative control group received no chemotherapy; the positive control group received cyclophosphamide on day 9 post-depilation at a dose of 120 mg/kg and no preventive medication. All other treatment groups were treated with daily topical formulations from day 7 until day 14 post-depilation and received cyclophosphamide at a dose of 120 mg on day 9 post-depilation.

All animals in the negative control group showed observable growth of normal-color and -quality hair by day 12 (Figure 3). The hair continued to grow longer until it reached its normal length by day 16. Afterwards, the hair elongation stopped, and all of the mice appeared completely normal. By day 19, the skin color of the mice was back to pink, which denoted that the hair had started the telogen phase. By day 27, the skin color was still pink, indicating the continuation of the hair resting phase. All observed hair was completely normal in quality, color, and length until day 27.

Regarding the positive control animals, a delay in hair regrowth was observable in comparison to the negative control group (Figure 3). By day 14, the mice’s backs were black yet not covered in hair, and by day 15, short feathery hair started to appear. The hair quality was so poor that it could fall off by simple friction. By day 16, most of the hair fell off the mice’s backs, and grey skin was revealed. Afterwards, the hair started to regrow until it reached a normal length by day 27. Most of the regrown hair was white, and the poor hair quality was remarkable.

The PHB group showed moderate-length hair that covered most of the mice’s backs by day 12; however, the hair quality was poor relative to the negative control group, yet not as poor as that of the positive control group (Figure 3). By day 14, some of the back hair had fallen off; however, most of the back was still covered with hair in half of the animals of the group, while the other half showed low-density hair, better represented by HGI (Section 2.3.3. On day 16, hair regrowth was observable in all mice. The regrown hair’s quality and color were comparable to those of the negative control group by day 27, except for one animal whose hair color was grey.

The PGZ group showed black skin color on day 12, yet no shafts appeared (Figure 3). By day 14, tiny hair shafts appeared on the backs of the animals, which totally fell off by day 16, showing totally bare skin. By day 19, the hair had regrown to medium length, and it reached the normal mouse hair length by day 27. The regrown hair showed moderate quality and density, while the hair color was variable between animals. One animal appeared with totally white hair, while all of the others showed grey hair.

The RIF group gave the least promising results of all treatment groups (Figure 3). By day 12, all of the mice’s backs were still uncovered with hair and bare, except for a single animal whose skin showed a black color. By day 14, all of the mice were still uncovered with hair, and no differences from day 12 were observed. By day 19, the mice’s backs were black, yet no hair shafts appeared. The first regrown hair was observable by day 23. All of the hair at that time was feathery, white, and of poor quality, as shown in Figure 3. By day 27, the hair reached the normal length. The poor hair quality was noticeable, and most of the hair was white. In general, the RIF group results were comparable to those of the positive control group.

The NAC group showed remarkable hair growth in four out of six animals by day 12 (Figure 3). At that stage, the NAC group results were promising and comparable to those of the negative control group. By day 14, the hair reached normal length and moderate coverage in most animals, yet not all, better represented by HGI (Section 2.3.3). By day 16, the hair density declined, showing that some of the grown hair had fallen off. By day 19, the hair had regrown remarkably. By day 27, the hair reached full length, yet most of the hair was white. The NAC group showed very promising results until day 14 of the experiment, and then hair growth and quality showed an observable decline afterwards.

The TAC group showed moderate-length hair growth by day 12, followed by continuous hair growth at all time points (Figure 3). The hair density was mostly normal, except for some uncovered areas. However, the regrown hair’s quality was moderate, and its color was a mixture of black and white—an observation noticed on day 19, and which remained until day 27.

Most of the blank CS NPs groups showed results comparable to those of the positive control (Figure 3). Most of the mice did not regrow hair until day 23. The regrown hair was scarce and of poor quality. It fell off easily, and most of the mice’s backs were uncovered until day 27.

Conclusively, all of the treatment groups showed better hair quality and faster regrowth than the positive control group, except for the blank CS NPs group. None of the treatment groups was the same as the negative control group, yet some were comparable to it. The NAC and TAC groups were very promising during the first part of the study (until day 14), and then a decline in hair density and quality was observable. The PGZ group did not show fast hair regrowth, but the regrown hair was of better quality than that in groups 3, 4, 5, and the positive control. The PHB group presented the most promising results, where the hair regrowth speed was comparable to that of the negative control group, and most of the animals showed good hair quality and color by the end of the study. The difference between the PHB group and the negative control appeared as poorer hair density of the PHB group during days 10 to 23 of the study. The RIF group was the least promising of all of the treatment groups. The results of the RIF group were comparable to those of the positive control in terms of hair regrowth speed and the quality and color of the regrown hair. The blank CS NPs group showed very poor hair and slow hair recovery, as well as no protection against the cytotoxic insult to HFs. It is worthwhile to mention that individual variations were observable in each group; however, the aforementioned observations are those that describe the hair growth behavior of most animals in each group. On another note, all of the treatment groups were observed until day 35 p.d., and no remarkable differences from day 27 were noticed.

#### 2.3.3. Hair Growth Index Score

**Figure 4:**
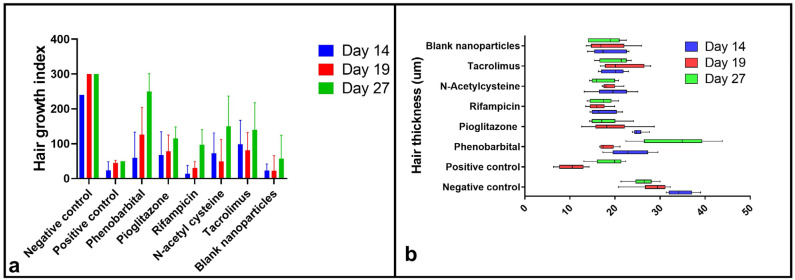
(**a**) Hair growth index score; data are presented as the mean ± SD. (**b**) Hair thickness in µm, measured under scanning electron microscope; data are represented as boxplots, where the horizontal line within each box indicates the median, the box edges represent the interquartile range (IQR), and the whiskers denote the full data range. The negative control group received no chemotherapy; the positive control group received cyclophosphamide on day 9 post-depilation, at a dose of 120 mg/kg, and no preventive medication. All other treatment groups were treated with daily topical formulations from day 7 until day 14 post-depilation and received cyclophosphamide at a dose of 120 mg/kg on day 9 post-depilation. Note that the positive control group’s hair thickness on day 14 is missing due to the absence of any hair for collection.

The HGI was calculated at three distinct time points: days 14, 19, and 27 p.d. (Figure 4a). The selection of these time points was based on the approximate continuation of the anagen VI phase, the onset of the catagen phase, and the onset of the telogen phase in C57BL/6 mice’s hair cycle [70].

On day 14, the negative control group’s score was 240.0 ± 0, and that of the positive control group was 23.33 ± 25.17. The TAC group showed the highest HGI of all treatment groups (98.33 ± 69.04), followed by the NAC group (72.50 ± 58.29), PGZ group (67.50 ± 66.76), PHB group (59.17 ± 74.32), RIF group (13.33 ± 24.22), and blank CS NPs group (22.86 ± 18.90). The difference among groups was significant (*p* ≤ 0.05), and multiple comparisons showed that the negative control group had a significantly higher HGI than all other groups (*p* ≤ 0.05), whereas the differences between the positive control and treatment groups were all non-significant (*p* > 0.05).

On day 19, the negative control group’s score was 300.0 ± 0, and that of the positive control group was 45.0 ± 7.07. The PHB group’s results were the highest among the treatment groups (126.0 ± 78.60), followed by the TAC group (81.00 ± 51.04), PGZ group (78.00 ± 47.12), NAC group (49 ± 67.79), RIF group (30.00 ± 18.70), and blank CS NPs group (22.00 ± 43.82). The difference among groups was significant (*p* ≤ 0.05). Similar to day 14, the negative control group showed a significantly higher HGI than all other groups (*p* ≤ 0.05). Multiple comparisons between the positive control and treatment groups were all non-significant (*p* > 0.05).

On day 27, the negative control group’s score was 300.0 ± 0, and that of the positive control group was 50.0 ± 0. The PHB group’s results were the highest among the treatment groups (250.00 ± 50.99), followed by the NAC group (150.00 ± 86.70), TAC group (140.00 ± 77.78), PGZ group (115 ± 33.17), RIF group (97.50 ± 42.72), and blank CS NPs group (57.50 ± 66.50). The difference among the five drug-treated groups was significant (*p* ≤ 0.05). Multiple comparisons showed that the PHB group had a significantly higher HGI than both the PGZ and RIF groups (*p* ≤ 0.05). Meanwhile, all other comparisons were statistically insignificantly different (*p* > 0.05).

**Figure A2:**
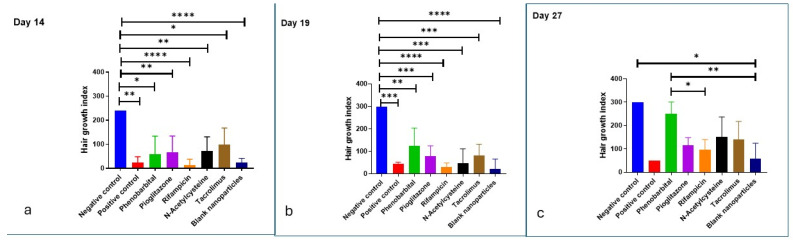
Hair growth index score on days 14, 19, and 27 (**a**–**c**); the negative control group received no chemotherapy, the positive control group received cyclophosphamide on day 9 post-depilation at a dose of 120 mg/kg and no preventive medication. All other treatment groups were treated with daily topical formulations from day 7 till day 14 post-depilation and received cyclophosphamide at a dose of 120 mg/kg on day 9 post-depilation. Note that the positive control group hair thickness on day 14 is missing due to the absence of any hair for collection. * *p* < 0.05; ** *p* < 0.01; *** *p* < 0.001; **** *p* < 0.0001.

The detailed statistical analysis of HGI is displayed in Figure A2.

#### 2.3.4. Hair Examination by Scanning Electron Microscope and Hair Thickness Measurement

**Figure 5:**
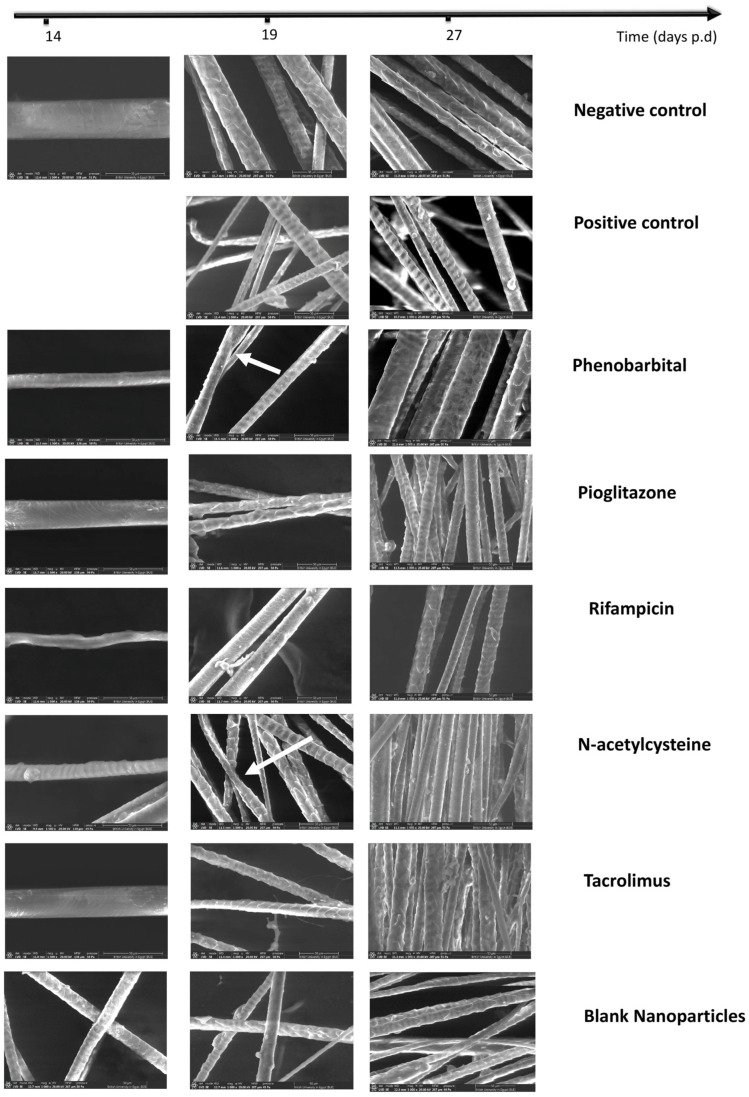
Scanning electron microscope examination of collected hair specimens on days 14, 19, and 27 post-depilation (from left to right, respectively): the negative control group received no chemotherapy; the positive control group received cyclophosphamide on day 9 post-depilation, at a dose of 120 mg/kg, and no preventive medication. All of the other treatment groups were treated with daily topical formulations from day 7 to day 14 post-depilation and received cyclophosphamide at a dose of 120 mg/kg on day 9 post-depilation. Note that the positive control group image for day 14 is missing due to the absence of any hair for collection. Note the complete absence of the normal ridged pattern of hair in the rifampicin group on day 14. The white arrows show abnormal bulging of the hair on day 19 in the phenobarbital and N-acetylcysteine groups.

Hair specimens collected at selected time points (days 14, 19, and 27 p.d.) were examined under SEM (Figure 5), and the hair thickness was measured (Figure 4b). On day 14, upon examining the hair by SEM, the positive control sample was missing due to the lack of hair for collection at that time point. Particularly, the RIF group’s hair showed very poor quality, with abnormal curvatures and a complete absence of ridges. On day 19, the hair of the positive control group was short, yet with normal ridges. The PHB hair showed an abnormal bulge, and the RIF hair was very short with minimal ridges. All of the other treatment groups were comparable to the negative control. By day 27, the hair of all groups was comparable to that of the negative control. Bending of hair from the positive control group was observable on day 19, which was restored on day 27 (Figure 5). Bulging of hair shafts in the PHB group was detected on day 19, which was restored on day 27 (Figure 5). The RIF group showed complete absence of the characteristic ridged pattern of hair, along with bending and an irregular structure, on day 14, which was restored on days 19 and 27 (Figure 5). The presence of indentations was observed in the NAC group on day 14; the structure was restored on days 19 and 27 (Figure 4). All of the aforementioned observations indicate defective cuticle formation of hair [71,72]. The blank CS NPs group’s examined hair was of a normal structure (Figure 5).

The hair thickness measurements (Figure 4b) revealed that, on day 14, the hair thickness scores were in the following order: negative control (34.55 µm ± 2.84), PGZ group (25.11 µm ± 1.36), PHB group (23.22 µm ± 4.53), TAC group (19.71 µm ± 2.58), NAC group (19.45 µm ± 4.15), blank CS NPs group (18.35 µm ± 3.68), and RIF group (17.35 µm ± 2.90). One-way ANOVA showed a significant difference (*p* < 0.05) among groups. Multiple comparisons showed a significant difference between the negative control and each of the treatment groups (*p* ≤ 0.05). Both the PHB and PGZ groups’ hair samples were significantly thicker than those of the RIF group (*p* < 0.05). Also, the PGZ group’s hair was significantly thicker than that of the blank CS NPs group (*p* < 0.05). All other comparisons were statistically non-significantly different (*p* > 0.05).

On day 19, the hair thickness scores were in the following order: negative control (28.57 µm ± 4.03), TAC group (21.51 µm ± 4.51), PGZ group (19.06 µm ± 5.34), NAC group (18.68 µm ± 1.78), blank CS NPs group (18.18 µm ± 4.50), PHB group (18.09 µm ± 1.75), and positive control (10.39 µm ± 2.88). One-way ANOVA showed a significant difference (*p* < 0.05) among groups. Multiple comparisons showed that the negative control group was significantly thicker than the positive control and each of the treatment groups (*p* < 0.05). Groups treated with PHB, PGZ, NAC, TAC, and blank CS NPs showed significantly thicker hair than the positive control group (*p* < 0.05). All other comparisons were statistically non-significantly different (*p* > 0.05).

On day 27, the hair thickness scores were in the following order: PHB group (33.62 µm ± 7.54), negative control (26.25 ± 2.86), TAC group (20.18 µm ± 3.22), positive control (18.89 µm ± 3.40), blank CS NPs group (18.17 µm ± 3.50), PGZ group (17.72 µm ± 3.50), RIF group (17.15 µm ± 2.62), and NAC group (16.96 µm ± 2.67). One-way ANOVA showed a statistically significant difference (*p* ≤ 0.05) among groups. Multiple comparisons showed that the PHB group’s hair was significantly thicker than that of all of the other groups (*p* < 0.05). The negative control hair was significantly thicker than that belonging to the positive control group (*p* < 0.05) and each of the PGZ, RIF, NAC, and blank CS NPs groups (*p* < 0.05). All other comparisons were statistically insignificantly different (*p* > 0.05).

**Figure A3:**
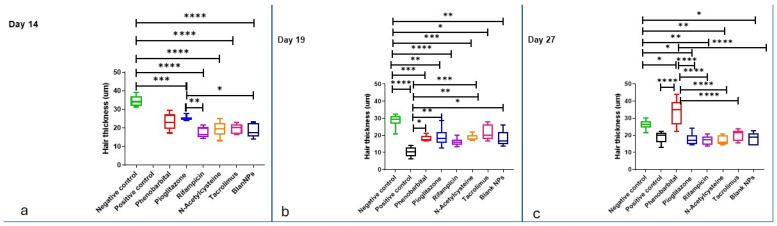
Hair thickness in µm measured under scanning electron microscope on days 14, 19, and 27 (**a**–**c**); the negative control group received no chemotherapy, the positive control group received cyclophosphamide on day 9 post-depilation at a dose of 120 mg/kg and no preventive medication. All other treatment groups were treated with daily topical formulations from day 7 till day 14 post-depilation and received cyclophosphamide at a dose of 120 mg/kg on day 9 post-depilation. Note that the positive control group hair thickness on day 14 is missing due to the absence of any hair for collection. Data is represented as box plots, where the horizontal line within each box indicates the median, the box edges represent the interquartile range (IQR), and the whiskers denote the full data range * *p* < 0.05; ** *p* < 0.01; *** *p* < 0.001; **** *p* < 0.0001.

The detailed statistical analysis of hair thickness is displayed in Figure A3.

#### 2.3.5. Histopathological Examination

**Figure 6:**
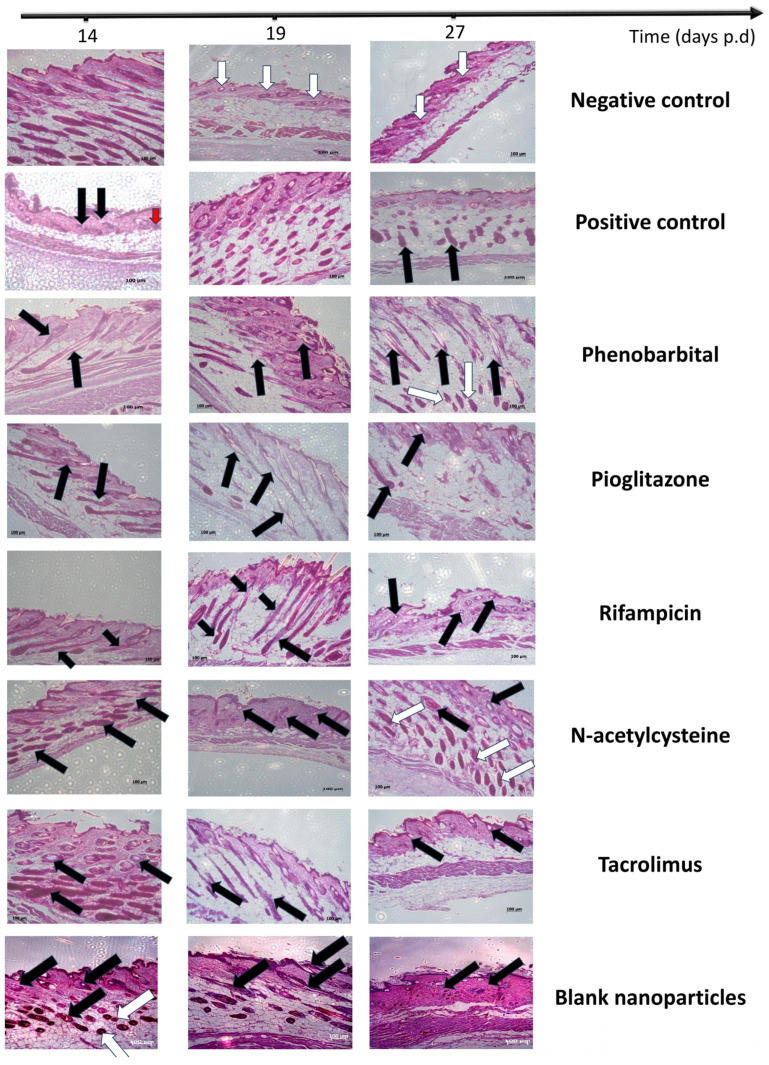
Histopathological examination of collected skin specimens on days 14, 19, and 27 post-depilation (from left to right, respectively): the negative control group received no chemotherapy; the positive control group received cyclophosphamide on day 9 post-depilation, at a dose of 120 mg/kg, and no preventive medication. All other treatment groups were treated with daily topical formulations from day 7 until day 14 post-depilation and received cyclophosphamide at a dose of 120 mg/kg on day 9 post-depilation (magnification power 100×). Note that the white arrows indicate normal hair follicles, while black arrows indicate dystrophic hair follicles with wide-open hair canals, irregular shapes, or no hair shafts. The red arrow in the positive control group on day 14 shows the presence of an orphan sebaceous gland.

**Figure 7:**
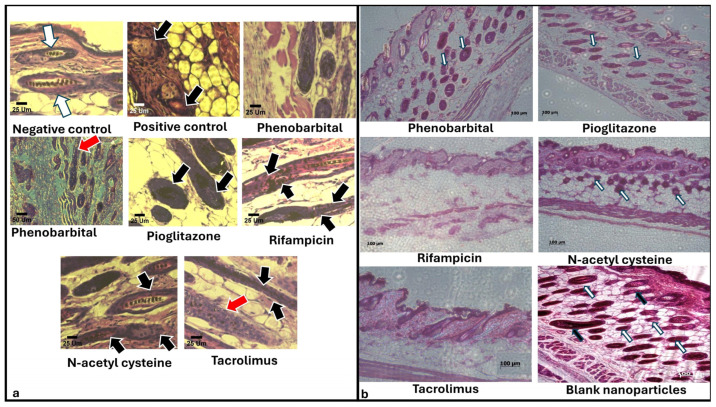
(**a**) Histopathological examination of collected skin specimens on day 14 post-depilation (magnification power 200×). (**b**) Histopathological examination of collected skin specimens on day 35 post-depilation (magnification power 100×). White arrows indicate normal-structure HFs. Red arrows indicate the unorganized melanin production within the hair shafts due to the lack of a typical zebra structure. Black arrows denote the presence of ectopic melanin granules within the inner root sheath of the hair canal. Note the signs of secondary recovery: the presence of anagen hair, and normal HFs within the subcutis (white arrows) in the phenobarbital, pioglitazone, and N-acetylcysteine groups on day 35.

The collected skin samples on days 14, 19, and 27 were microscopically examined to detect the changes occurring in the hair growth cycle (Figure 6 and Figure 7).

The negative control group represented the normal histology of a hair cycle after depilation-induced anagen. On day 14, the negative control group sample was in a late anagen phase, probably anagen VI. This could be detected by the presence of a large number of hair bulbs deep within the subcutaneous layer above the pannus carnosus muscle, as well as by the emergence of hair shafts through the epidermis (Figure 6) [70]. Upon closer examination of hair bulbs and shafts (Figure 7a), we detected that they appeared to be of normal structure. The hair shafts possessed the typical zebra assembly with melanin granules, and the hair bulbs appeared normal. The sebaceous gland appeared to be of normal size and structure, and there were no signs of HF dystrophy. By day 19, the negative control hair had already reached the catagen or telogen phase, where all hair bulbs reside within the dermis, with normal hair bulb and shaft structure (Figure 6). We may conclude that the catagen phase took place at a time between day 14 and day 19, which is within the normal range [70]. By day 27, the mouse hair was still in the telogen phase, and no major changes were observable compared to day 19 (Figure 6).

The examination of the positive control group revealed that the chemotherapeutic insult led the mice’s hair into a dystrophic catagen pathway. By day 14, the number of HFs within the collected specimen was minimal. The observed HFs were all within the dermis, which indicates a telogen phase due to the sudden termination of anagen, in addition to the presence of an orphan sebaceous gland, which denotes the apoptosis of its accompanying HF (Figure 6) [69]. Upon examining sections with a higher magnification lens, the presence of dystrophic telogen HFs was noticeable due to the appearance of a hair canal without club hair within the dermis, in addition to the appearance of an orphan sebaceous gland (Figure 7a) [69]. By day 19, the positive control reached the secondary recovery after dystrophic catagen and shortened telogen stages [73]. This was revealed by the histopathological examination of the skin, which showed that the hair was in a normal anagen stage, and hair bulbs were located within the subcutis layer [70] (Figure 6). By day 27, many hair bulbs resided within the middle of the subcutis, denoting that the hair was still in a catagen phase. Some hair bulbs resided in the dermis layer, which reached the catagen or telogen phase. The general morphology of some hair bulbs appeared normal, while others within the mid-subcutis layer were irregular in shape, suggesting dystrophy even within secondary recovery (Figure 6).

By histopathological examination, we confirmed that all five used agents (PHB, PGZ, RIF, NAC, and TAC) and the blank CS NPs shifted the hair cycle damage to a dystrophic anagen pathway rather than a dystrophic catagen, as in the positive control. On day 14, all six groups were found to be in a late dystrophic anagen VI stage. This was detected by the appearance of twisted HFs, hair shaft fragments, and a widened distal hair canal (Figure 6). All of the hair bulbs were allocated deep within the subcutis above the pannus carnosus muscle. The aforementioned findings were also confirmed by higher-power magnification, as we detected disrupted hair shaft pigmentation and unorganized ectopic melanin granules within the inner root sheath of the hair canal (Figure 7a) [69,70].

On day 19, the differentiation of the groups was remarkable. Group 1 (PHB) and 2 (PGZ) HFs were categorized in two different stages: late dystrophic catagen, and dystrophic telogen (Figure 6). Groups 3 (RIF), 5 (TAC), and 6 (blank CS NPs) were all found to be in a late dystrophic catagen stage (Figure 6). The NAC group showed only dystrophic telogen (Figure 6). Late catagen hair bulbs are located in the middle of the subcutis, while telogen hair bulbs lie within the dermis. The HF dystrophy was confirmed by the appearance of widened hair canals and the occasional absence of club hair [69,70].

On day 27, the PHB group’s hair was found to be in three distinct stages: late dystrophic catagen, dystrophic telogen, and healthy anagen VI (Figure 6). As mentioned earlier, catagen hair resides in the middle of the subcutis or the borderline between the dermis and subcutis, while telogen hair resides in the dermis. Dystrophy was detected by the abnormally wide-open hair canals and the occasional absence of club hair. Late anagen HFs were found deep within the subcutis, with no signs of dystrophy. Similarly, the NAC group’s HFs showed two distinct stages: dystrophic telogen and healthy anagen VI (Figure 6). Therefore, we may conclude that by day 27, groups 1 (PHB) and 4 (NAC) had started the secondary recovery of HFs. Groups 2 (PGZ), 3 (RIF), 5 (TAC), and 6 (blank CS NPs) were all in the dystrophic telogen stage (Figure 6) [69,70].

To track the progress of secondary recovery, skin samples were collected on day 35 and examined. PHB group hair was categorized into three distinct stages: dystrophic telogen, healthy anagen, and healthy catagen (Figure 7b). Dystrophic telogen hair is allocated within the dermis, with wide hair canals and, often, no club hair. Healthy HFs look normal from a transverse section view. Catagen hair resides in the mid-subcutis, and anagen hair resides deep in the subcutis. Similarly, groups 2 (PGZ), 4 (NAC), and 6 (blank CS NPs) showed both dystrophic telogen and healthy anagen hair (Figure 7b). A comparative summary of the findings of the in vivo study is presented in Table 1. 

## 3. Discussion

In this study, we evaluated the effects of five different agents, individually administered topically, for the prevention and/or treatment of CIA. To enhance their protective effects, the agents were encapsulated into CS NPs to optimize HF targeting, as polymeric nanoparticles have been reported to preferentially deposit within HFs upon topical application [27]. CS was specifically chosen due to its previously demonstrated hair-growth-promoting properties in various types of alopecia. Additionally, its tissue-regenerative capabilities make it a suitable candidate for drug delivery in our study [29,74,75,76,77]. In addition, a previous finding reported the ability of CS NPs to enhance the follicular deposition of minoxidil by six times the drug solution [34]. The successful preparation of drug-loaded CS NPs was confirmed through DLS (Figure 1). Morphological examination of CS NPs by TEM showed spherical particles ~200 nm in size (Figure 2a). FT-IR spectroscopy further verified successful drug encapsulation, as the characteristic peaks of each drug disappeared in the spectra of the CS NPs (Figure 2b,c).

First, as regards the selected formulation findings and correlated physicochemical findings, we employed a modified ionic gelation method—a simple, reproducible, and organic solvent-free technique—to prepare CS NPs loaded with the target drugs. However, optimizing the formulation technique is necessary to achieve the optimal PS and EE% for each drug. In this study, we maintained all formulation parameters constant to accurately assess the in vivo effects of each drug in preventing and/or treating CIA. The formulations (F1–F4) exhibited no significant differences in PS (Figure 1), suggesting that the drug type and nature had no substantial impact on the CS NPs’ size. However, the PS of F5 was significantly lower (*p* < 0.05), as its formulation had been previously optimized to enhance PS and EE% through specific modifications [52]. Other formulations were prepared under the same conditions to ensure consistency and allow for reliable efficacy comparisons.

All of the formulations were cationic, as expected due to the positively charged CS polymer. The polyanion TPP was used at a concentration that neutralized some of the CS’s positive charges while maintaining enough charge to stabilize the nanoparticles and prevent aggregation [78]. Using TPP in higher concentrations may omit all positive charges, leading to complete aggregation and system failure. All of the used drugs have no net charge [79,80,81,82,83]. Hence, all of the formulations kept a positive surface charge.

The EE% and DL% of the PHB formulation (F1) could have been maximized, while maintaining the PS within an acceptable range for the desired targeting area, by further optimizing the ratio between the co-solvent phase and the aqueous phase. This interpretation can be attributed to the fact that PHB exhibits preferential solvation in both water-rich mixtures (due to interaction with water molecules by hydrophobic hydration) and aqueous co-solvent-rich mixtures (due to the basic behavior of co-solvents) [84]. To the best of our knowledge, PHB-loaded CS NPs have not yet been studied by researchers. CS NPs encapsulating PHB could be a beneficial system for using PHB as an anticonvulsant while encapsulated in CS NPs to achieve brain targeting, as CS NPs have been reported to be a good carrier system for brain targeting via the intranasal route [85].

The EE% and DL% of PGZ HCl could have been maximized by adding PEG 400 to create a ternary mixture of water, PEG 400, and propylene glycol at specific ratios [86]. Enhanced solubility of PGZ in the specific mixture is expected; hence, further investigation of using the co-solvent mixture is recommended [86]. PGZ was previously formulated by researchers in CS/PEG blended PLGA nanoparticles by the solvent evaporation technique; a PS of 323 ± 1.15 nm and an EE of 61.7 ± 2.91% were reported [87]. PGZ was also co-loaded with curcumin in CS NPs using the ionic gelation technique while adding polysorbate 80 and DMSO as co-solvents [88]. Moreover, PGZ was a candidate drug for repurposing for the treatment of Alzheimer’s disease [89], an interesting future perspective for using PGZ-loaded CS NPs.

As for RIF, it yielded a minimal EE% and DL% in our study (Figure 1). We assume that this is attributed to the poor solubility of RIF in aqueous media and many aqueous co-solvents, as well as its high solubility in acidic media [90]. Therefore, as a corrective step, we recommend increasing the acetic acid concentration and increasing the shear stress [91,92,93,94,95,96,97]. This was previously tested in a study by Rawal et al. [96], where the EE% reached 72%.

Regarding NAC, we assume that the low EE% value (Figure 1) can be attributed to the presence of the co-solvent (propylene glycol), because NAC is a hydrophilic drug [98]. Therefore, the presence of propylene glycol probably minimized the drug’s solubility due to the lower aqueous proportion in the formulation mixture. Moreover, the used CS concentration (0.1%) is minimal for conjugation with NAC itself in the presence of TPP. This is confirmed by the high DL% value, which provides evidence that the low amount of polymer was not suitable for the very high dose of drug. Thus, for further testing of NAC-loaded CS NPs, it is recommended to use a higher CS concentration (0.4%) without adding co-solvents. It is worthwhile to mention that NAC was previously formulated in CS and CS derivatives [99,100,101], in addition to being used as a delivery system itself in conjunction with polymers [102,103,104,105]. It was also reported that NAC binds to CS via the thiol group, yielding thiolated CS NPs [101].

Concerning TAC-loaded CS NPs, the EE% and DL% values obtained were similar to those of our previous study [52]. In brief, the use of a 5:2:3 ratio was optimal for drug entrapment to form nanoparticles with an acceptable ratio between polycations and polyanions, along with the amount of co-solvent. The solvent selection was based on a comparative study between PG, PEG 400, and ethylene glycol (EG), in which EG showed a higher EE% yet a larger PS. The CS concentration of 0.1% was selected because it provided superior DL% compared to higher CS concentrations, probably due to the higher viscosity of CS solution at higher concentrations, which decreases the dispersion of CS; in turn, this deters the drug loading into the polymer during the nanoparticle formation process [106,107].

Second, to find out the potential of the synthesized drug-loaded CS NPs as a protective and/or therapeutic regimen for CIA, we need to understand the normal physiology of hair. The hair growth cycle is divided into three main distinct phases: anagen, catagen, and telogen. The anagen phase is defined as the growth phase of the hair in which the HFs are in active mitosis. The catagen phase is the resting phase, in which the HFs start apoptosis and regression. The telogen phase is a complete resting of the HFs, in which no hair growth occurs [108]. Most adult animal hair is normally in the telogen (resting phase) at any given time point, while most adult human scalp hair is in the anagen (growing phase) at any time point [70,109]. The underlying pathology of CIA is explained as the chemotherapeutic insult causing instant severe damage to anagen HFs’ cells, which varies according to the used cytotoxic agent, dose, and individual response [19]. This follicular dystrophy is usually manifested as one of two pathways: either dystrophic catagen or dystrophic anagen. The dystrophic catagen pathway is considered to cause more severe cellular damage than the dystrophic anagen pathway. During the dystrophic catagen pathway, a sudden cessation of the anagen phase occurs, which leads to hair shedding and the start of a shortened dystrophic telogen phase. Afterwards, secondary recovery of hair is observable by the production of new healthy anagen hair that grows into a normal catagen and then telogen hair cycle. The dystrophic anagen pathway is characterized by minimized HF damage. During dystrophic anagen, the hair experiences a primary recovery after hair shedding, in which the hair undergoes dystrophic anagen, dystrophic catagen, and dystrophic telogen successively. Afterwards, secondary recovery occurs, and a normal hair cycle starts with a healthy anagen, followed by catagen and telogen. It is worth mentioning that dystrophic anagen hair may shift to dystrophic catagen during different hair growth cycle stages [69,73]. Chemotherapeutic insult affects only anagen hair that exhibits mitotic division [60]. In order to assess CIA prevention, anagen VI should be induced in adult mice via a depilation process [110].

Our findings from the in vivo study of the management of CIA by topical application of CS NPs loaded with PHB, PGZ, RIF, NAC, or TAC revealed that the macroscopic results were correlated with the microscopic observations in each of the treatment and control groups However, the findings determined from the blank CS NPs group showed minimal regrown hair throughout the study, despite anagen hair being observed in skin specimens by microscopic examination. The negative control group of animals showed a completely normal hair regrowth cycle post-depilation (Figure 3). By day 14, the negative control group reached a healthy late anagen phase (Figure 4a and Figure 6). By day 19, all of the negative control animals had already reached telogen, which suggests that catagen took place at a time point between days 14 and 19, most probably on day 16, denoted by the mice’s skin color (Figure 3 and Figure 6). The described post-depilation hair regrowth pattern and timeline were completely normal [70]. Thus, they were used as a reference to detect pathophysiological changes in the positive control and treatment groups. Upon interpretation of all macroscopic and microscopic results of our study, we may conclude that all five agents provided a certain degree of protection from the cytotoxic agent (CYP). This was demonstrated by the fact that the positive control group had undergone a dystrophic catagen pathway, while all six treatment groups experienced a dystrophic anagen. A 120 mg/kg dose of CYP was controversially reported to induce dystrophic anagen or dystrophic catagen in different studies [69,73]. However, in the current study, our findings provide evidence that the 120 mg/kg dose induced dystrophic catagen. This was confirmed by the complete absence of macroscopically visible hair shafts until day 14 of the study (Figure 3 and Figure 4a) and the observation of dystrophic telogen hair on day 14, with the complete absence of late anagen hair (Figure 6 and Figure 7a). The start of secondary recovery was observable by day 19 in the positive control group, as revealed by hair regrowth (Figure 3 and Figure 4a) and the appearance of mid-anagen hair in the middle of the subcutis layer (Figure 6). By day 27, a catagen stage was observable, demonstrated by the presence of normal-growing hair that almost reached a normal length (Figure 3 and Figure 4a) and the presence of HFs in the mid-subcutis layer (Figure 6). The poor quality of the regrown hair was still observable upon secondary recovery (Figure 3, Figure 4a, and Figure 5).

All of treatment groups underwent a dystrophic anagen pathway, as demonstrated by the appearance of poor-quality hair by day 14 and the dystrophic anagen stage within the primary recovery, followed by dystrophic catagen and telogen stages. This finding proves that all five agents exerted a degree of protection against CIA. However, this protection was incomplete, as none of the treatment groups showed a normal hair growth pattern, comparable to the negative control. The main difference between treatment groups is the speed at which the appearance of signs of secondary recovery occurs. Blank CS NPs resulted in limited visible hair regrowth. However, microscopic examination revealed the presence of dystrophic anagen follicles, as indicated by the detection of anagen-phase hair at all assessed time points. This suggests that CS may exert regenerative effects by promoting mitotic activity within HFs, thereby partially stimulating hair regeneration [29]. Nonetheless, the observed damage to follicular cells likely impeded the progression of these newly formed hairs to a fully developed stage, preventing the emergence and retention of visible hair shafts on the skin surface.

The PHB group was the fastest of all treatment groups in terms of recovery. The regrown mouse hair appeared normal and comparable to the negative control group’s hair (for some animals) starting from day 27 (Figure 3 and Figure 4a,b). The appearance of normal late anagen HFs was microscopically observable on day 27 (Figure 6) and continued to a normal catagen by day 35 (Figure 7b). This may be explained by the fact that PHB is an activator of the constitutive androstane receptor (CAR) via the inhibition of epidermal growth factor receptor (EGFR) signaling [111]. CAR is a substrate of ABCB1 (angiotensin-binding cassette B1), one of the ABC family of transporters responsible for multidrug resistance by efflux of anticancer drugs, a mechanism of action that leads to failure of cancer treatment if the medication is administered systemically [43]. ABCB1 was found to be widely expressed within both human and mouse HFs, with a higher expression within mouse HFs [46,112]; thus, we declare that the incorporation of PHB into HF-targeted drug delivery systems is a very promising approach for the prevention of CIA.

Second in terms of recovery pace was the NAC group. Although the hair quality of the NAC group was inferior to that of groups 2 (PGZ) and 5 (TAC) by days 27 and 35 (Figure 3 and Figure 4a,b), by histopathological examination, it was evident that the NAC group started the primary recovery on day 27. This was concluded upon finding healthy anagen HFs within the subcutis of the examined specimens (Figure 6), which continued their progression by day 35 (Figure 7b). NAC is a prodrug for glutathione, a mechanism of action by which it is famous for being an antidote to paracetamol [113]. Similarly, glutathione was reported to cause drug resistance to alkylating agents on a cellular level via conjugation that produces inactive conjugates [114]. This interpretation is in agreement with the previous findings of Jimenez and his team [9]. This demonstrated that NAC was very efficacious in the prevention of CYP-induced CIA upon administration both systemically and topically via subcutaneous injection, yet it was ineffective against cytarabine-induced CIA. Thus, we may conclude that NAC would be suitable for the prevention of CIA that results from alkylating agents only. Intuitively, systemic administration of NAC would impair the course of cancer treatment by alkylating agents, a previously reported finding [115]. Thus, the topical route is much preferable, preferentially using an HF-targeting system.

Third in speed was the PGZ group, which produced the best hair quality during the primary recovery stage (Figure 3 and Figure 4a,b), with the least signs of dystrophy (Figure 6), but it was slower than groups 1 (PHB) and 4 (NAC) in the start of the second recovery phase (Figure 3 and Figure 4a,b), which were minimally detected microscopically by day 27 (Figure 6) and were fully detectable by day 35 (Figure 7b). PGZ is an agonist to peroxisome proliferation receptor γ (PPAR γ), a receptor that was reported to be found within the sebaceous duct and erector pili muscle of HFs of both humans and mice [116,117]. It was also proven to play a role in HF development and stem cell physiology. The upregulation of PPAR γ was reported to protect against CYP-induced hepatotoxicity, as it was also proven to be a cause of drug resistance that leads to anticancer failure [118,119]. To the best of our knowledge, there is no comparison between the mRNA expression of PPAR γ in mice vs. humans.

Both groups 3 (RIF) and 5 (TAC) showed no signs of secondary recovery until day 35 (Figure 6 and Figure 7b), yet the TAC group showed better hair quality in comparison to the RIF group throughout the study (Figure 3, Figure 4a,b, and Figure 5). It was remarkable in the RIF group particularly that most of the recovered hair was white (Figure 3 and Figure 4a). This reveals the predominance of ectopic melanin production, as confirmed by microscopic examination of the HFs and hair shafts, revealing the lack of the well-known zebra structure of hair shafts and the appearance of melanin granules outside their normal location inside the outer root sheath (Figure 7a) [69]. This observation was found in all treatment groups on day 14 of the study (Figure 6 and Figure 7a), yet the RIF group showed no progress in terms of the color of most regrown hair until day 27 (Figure 3 and Figure 4a). As regards the TAC group, our findings are in agreement with previously reported findings that topical TAC does protect against CYP-induced HF dystrophy by shifting the hair pathway from dystrophic catagen to dystrophic anagen [120]. However, the exact mechanism was unexplained. Being an immunomodulatory that works by the inhibition of calcineurin phosphatase—which, in turn, downregulates the production of IL-1 and IL-6 [121]—we may conclude that TAC creates its protective effect by inhibition of the inflammatory pathway that occurs upon exposure of the HFs to cytotoxic agents. The described effect is similar to results obtained using cyclosporine, steroids, growth factors, interleukins, and AS101 (immunomodulator) for CIA prevention [11,12,14,58,69,122]. A probable additional explanation is that the vasoconstrictive properties of TAC could hinder cytotoxic agents’ absorption within the target cells [57]—a mechanism similar to scalp cooling [6].

Finally, the RIF group unexpectedly yielded the least promising results, in terms of hair regrowth speed and regrown hair quality (Figure 3, Figure 4a,b, and Figure 5), throughout the study—a finding that was confirmed by microscopic examination (Figure 6 and Figure 7a). By day 35, no signs of secondary hair recovery were microscopically detectable (Figure 7b). RIF is a substrate of pregnane X receptor (PXR) that is responsible for the expression of ABCB1 [46], a mechanism of action very similar to that of PHB, but there is a major difference in protective efficacy. This could be attributed to the fact that the relative expression of mRNA of PXR in mice is lower than that in humans, whereas that of CAR is higher in mice than in humans; however, upon comparing the mRNA expression of the CAR receptor to that of the PXR receptor, it was found that CAR receptor expression is higher than that of the PXR receptor in both humans and mice [46]. Despite this, as a future perspective, we suggest comparing the efficacy of PHB vs. RIF on a human scalp cell line to get more reliable results. Another probable contributing factor to the inefficacy of RIF is the very low EE% in the synthesized formulation; thus, manipulation of the RIF formulation could enhance efficacy by better targeting the drug into HFs.

It is worthwhile to mention that hair dystrophy and CIA develop in a rising and falling arrangement, and the differentiation between HF stages is not constant for all adjacent hair [123]. Thus, the expressed results and interpretation resemble the majority of the examined hair and skin specimens.

SEM examination of hair is not a commonly used test in CIA studies; however, studies have reported that dystrophic HFs produce distorted hair, without the normal formation of the outer sheath cuticle [69,71]. Hair thickness was also previously reported to be a marker for the quality of regrown hair after the treatment of alopecia [124]. Therefore, we conducted an SEM examination of hair specimens in our study to determine the quality of regrown hair and differences between treatment groups (Figure 4b and Figure 5). The poor hair quality of all treatment groups by day 14 is correlated with being in a dystrophic anagen phase (primary recovery), while the lack of any regrown hair for collection in the positive control group denotes the dystrophic catagen. By day 19, we observed that all treatment groups except for RIF showed improved hair quality, but not the same as the negative control. The RIF group showed unridged short hair, which could be explained as a delayed recovery, whereas the hair of the positive control group looked normal yet very short, as they were in a primary recovery stage of dystrophic catagen. By day 27, all hair appeared normal and comparable to the negative control.

## 4. Materials and Methods

### 4.1. Materials

CS (Molecular weight 100,000–300,000 Da, degree of deacetylation 90%) and polysorbate 80 were purchased from ACROS ORGANICS (Fair Lawn, NJ, USA). TAC USP (Biocon, Electronics City, Bangalore, India) and pioglitazone HCl were kind gifts from Al Andalous for pharmaceutical industries (Giza, Egypt). PHB BP 2018/USP 41 and RIF compacted BP (Hebei Xingang pharmaceuticals, Shijiazhuang, China) were granted by the Egyptian International Pharmaceutical Industries Company (EIPICO, 10th of Ramadan City, Egypt) and Hikma Pharmaceuticals (Giza, Egypt), respectively. NAC and pentasodium tripolyphosphate anhydrous (TPP) were purchased from Sigma Aldrich (Eschenstr. 5, Taufkirchen, Germany). Propylene glycol (Batch No.: P75664) was purchased from ALPHA CHEMIKA (Mumbai, India). Orthophosphoric acid, sodium phosphate monobasic, acetonitrile, and methanol were of HPLC grade and purchased from Fisher Scientific (Fair Lawn, NJ, USA). Glacial acetic acid was purchased from Nile Pharmaceutical Company (Cairo, Egypt). Cyclophosphamide 500 mg (Endoxan™, Baxter Healthcare, Deerfield, IL, USA) was purchased from a local pharmacy.

### 4.2. Methods

#### 4.2.1. Preparation of Drug-Loaded Chitosan Nanoparticles

CS NPs loaded with PHB, PGZ, RIF, NAC, or TAC were prepared using an ionic gelation method adapted from our previous work [52] and based on the initially described protocol [78]. TPP was used as a crosslinker at a concentration of 0.05% *w*/*v* in an aqueous solution. Propylene glycol served as a co-solvent and was added to the aqueous TPP mixture under continuous magnetic stirring at 500 rpm. Meanwhile, CS was dissolved at a concentration of 0.1% *w*/*v* in 1% acetic acid (pH = 3). The TPP–co-solvent mixture was then added dropwise into the CS solution using a syringe, under vigorous magnetic stirring at 1000 rpm. The formulation was stirred for 30 min before being collected for further characterization.

Each drug was dissolved either in the TPP–co-solvent mixture or the CS solution, depending on its solubility profile. In some cases, heating at 60 °C and bath sonication (Elmasonic S60 H, Elma-Hans Schmidauer, Singen, Germany) were applied to facilitate dissolution. The selection of the solvent phase for each drug was guided by its physicochemical properties: PHB (logP = 1.5, PKa = 7.4) [125], RIF (logP = 3.7 and Pka = 1.7, related to the 4-hydroxy group/Pka = 7.9, related to 3-piperazine nitrogen) [126], and TAC (logP = 3.3, PKa= 9.96) [127] were added to the TPP–co-solvent mixture, whereas PGZ HCl (logP = 2.3, Pka = 5.8) [128] and NAC (logP = 0.696 and PKa = 8.3) [129] were added to the aqueous CS solution. Each drug was incorporated at concentrations optimized based on effective doses reported in the literature [9,52,130,131,132]. The CS/co-solvent/TPP ratio was maintained at 5:3:2 across all formulations. The aforementioned formulation parameters were adopted from a previous study by our team, in which TAC-loaded CS NPs were prepared and optimized [52]. Detailed compositions of the formulations are provided in Table 2.

#### 4.2.2. Nanoparticles’ Characterization

The freshly prepared drug-loaded CS NPs were diluted with deionized water (1:10 *v*/*v*) to avoid multiple scattering effects before measurement. The average PS, PDI, and ZP were determined using dynamic light scattering (DLS) and Laser Doppler Micro-electrophoresis, via a Zetasizer Nano ZS (Malvern Instruments, Worcestershire, UK). All measurements were performed at 25 °C and a scattering angle of 173° [133].

The PS distribution was reported as an intensity-weighted average, which is most sensitive to larger particles and aggregates. Each measurement represents the mean ± standard deviation from three independent replicates (*n* = 3). The PDI values were used to assess the width of the size distribution. The zeta potential values were obtained to estimate the surface charge and colloidal stability of the nanoparticles [134,135].

The collected colloidal suspension was initially centrifuged at 1000 rpm for 10 min at 25 °C to remove excess precipitates. The precipitate was discarded, and the resulting supernatant was subjected to a second centrifugation (Centurion Ltd.^®^ cooling centrifuge, Centurion Scientific Limited, West Sussex, UK) at 15,000 rpm for 1 h at 4 °C [136]. The supernatant was then discarded, and the pellet was collected for further analysis.

To extract the encapsulated drug, 2 mL of the respective mobile phase (Table 2) was added to the pellet, followed by mixing for 30 min in a bath sonicator (Elmasonic S60 H, Elma-Hans Schmidauer, Singen, Germany). The resulting solution was filtered through a 0.2 µm syringe filter, and 10 µL of the filtrate was injected into a Thermo Scientific^®^ Ultimate 3000 ultra-performance liquid chromatography (UPLC) system (Thermo Fisher Scientific, Waltham, MA, USA) for quantitative drug analysis. An autosampler was employed for precise quantification, with the chromatographic conditions for each formulation detailed in Table 3 [137,138,139,140,141,142,143,144,145,146,147].

The EE% and DL% were calculated using the following equations, respectively [148,149]:

$$EE\%=\left(\frac{Entrapped\;drug\;amount}{Total\;drug\;amount}\right)\times 100$$

$$DL\%=\left(\frac{Weight\;of\;entrapped\;drug}{Weight\;of\;nanoparticles}\right)\times 100$$

##### Microscopic Examination of Prepared Particles

The morphological characteristics of the prepared CS NPs were analyzed using a JEOL JEM-1010 TEM (Tokyo, Japan). To enhance visualization, a drop of 1% uranyl acetate was applied and allowed to air-dry before examining the sample under TEM [150].

##### Fourier-Transform Infrared Analysis (FT-IR)

The prepared CS nanosuspensions were analyzed for polyelectrolyte complex interactions using a Bruker^®^ RAM II FT-Raman spectrometer (Bruker Corporation, Billerica, MA, USA). To eliminate background interference in the FT-IR spectra of the CS NPs, approximately 0.2 mL of water was first applied to the diamond crystal to fully cover it, followed by a background scan. The water was then removed, and the same volume of nanoparticle suspension was placed on the crystal for scanning. For comparison, the FT-IR spectra of each incorporated drug (PHB, PGZ, RIF, NAC, and TAC) were also recorded alongside their respective drug-loaded nanoparticles. The analysis was conducted at room temperature, covering the infrared spectral range of 400–4000 cm^−1^, with a resolution of 4 cm^−1^ [151].

#### 4.2.3. In Vivo Comparison of the Efficacy of Chitosan Nanoparticles Loaded with Different Agents in the Prevention and/or Treatment of Chemotherapy-Induced Alopecia in an Animal Model

##### Animals

Forty-four female C57BL/6 mice (6–8 weeks old) were obtained from the AL-Fahd Company for Laboratory Animals (Giza, Egypt). This specific age, gender, and species were selected based on established CIA animal models [15,73,110,152,153,154,155]. Female mice were chosen to eliminate the potential influence of testosterone, which may induce hair loss through androgenic mechanisms [156].

The animals were housed at the British University in Egypt under controlled conditions, with a standard diet and ad libitum access to water. The ambient temperature was maintained between 25 and 28 °C using an air-conditioning system. The study was approved by the Research Ethics Committee at Ain Shams University (Approval number: ACUC-FP-ASU RHDIRB2020110301 REC#257), in compliance with the ARRIVE guidelines and the U.K. Animals (Scientific Procedures) Act, 1986. The mice were randomly assigned to eight groups: two control groups (negative and positive, *n* = 4 each) and six treatment groups (*n* = 6 each). Groups 1–5 received formulations F1–F5, respectively, and group 6 received blank CS NPs.

##### Induction of Anagen VI

Mice aged 6–8 weeks were selected to ensure that their HFs were in the telogen phase, as confirmed by the pink coloration of their skin [70,73,152,153,155]. To induce the anagen phase, hair on the dorsal region was removed using commercial wax strips (purchased from a local pharmacy in Egypt). This depilation procedure was performed under general anesthesia using an isoflurane inhalation anesthesia device.

##### Induction of Chemotherapy-Induced Alopecia

On day 9 post-depilation (p.d.), CIA was induced in all treatment groups and the positive control group via intraperitoneal injection of CYP at a dose of 120 mg/kg [73]. Endoxan™ powder was freshly dissolved in sterile 0.9% normal saline under aseptic conditions inside a laminar airflow cabinet (BioBase, Jinan, China). To enhance solubility, three drops of polysorbate 80 were added per 30 mL of the prepared solution [157].

##### Prevention and/or Treatment of Chemotherapy-Induced Alopecia

All of the treatment groups received a topical application of 1 mL of the corresponding formula (Table 3), whereas group 6 received blank CS NPs, on the depilated area, starting from day 7 p.d. until day 14 p.d. The drug concentration in each formula varied according to the reported dose: 225 µg/mL for PHB, 200 µg/mL for PGZ, 400 µg/mL for RIF, 30,000 µg/mL for NAC, and 300 µg/mL for TAC [9,52,130,131,132]. On day 9, the formulae were applied 2 h before the chemotherapeutic injection [59,73]. Both the positive and negative control groups did not receive any treatment. A representative mouse was euthanized from each group on days 14, 19, 27, and 35 p.d. by CO_2_ inhalation. Hair and skin specimens were collected for further investigation.

##### Evaluation of the Protective/Therapeutic Effects of the Formulae Against Chemotherapy-Induced Alopecia

- Observational analysis and hair growth index scoring

All of the mice were observed daily and photographed to detect visual changes in their skin color and hair growth patterns from day 0 (depilation day) until day 35 post-depilation. On days 14, 19, and 27, the hair growth index (HGI) score was recorded by two different persons, and the average score was calculated. The HGI calculation was adopted from Ref. [158]. The total score was obtained by multiplying the hair coverage score by the hair quality/color score. The hair coverage score ranged from 0 to 100% according to the observed hair-covered areas within the depilated mice’s backs [159]. The hair quality/color score was graded as 0, 1, 2, or 3 (0: no hair; 1: short broken white hair; 2: intermediate-length grey hair; 3: normal-length black hair). The total HGI score ranged from 0 (no hair) to 300 (completely normal hair).

- Scanning electron microscope examination

Hair samples were collected from a representative of each group on days 14, 19, and 27 p.d. for further examination. The collected hair was put on the imaging stub and examined using a Thermo^®^ Scientific field-emission scanning electron microscope (FESEM, Quattro S, Thermo Fisher Scientific, Waltham, MA, USA). The diameters of different hairs of each group were measured and recorded as an average of six measurements [124].

- Histopathological analysis

Skin samples were collected from representative mice of each group on days 14, 19, 27, and 35 p.d. after euthanizing the animals by CO_2_ inhalation. The collected samples were fixed in 10% formalin–saline for 24 h, and then washed with distilled water. Afterwards, the samples were dehydrated by washing with serial dilutions of methanol and ethanol. Subsequently, the samples were washed with absolute ethanol. The samples were put in xylene and then fixed in paraffin in a 56 °C oven for 24 h. The paraffin blocks were sectioned into 4 µm thick sections using a slide microtome. The sections were placed on a glass slide, deparaffinized, and then stained with hematoxylin and eosin [160]. The slides were examined by a light microscope (Axiostar plus, Carl Zeiss MicroImaging, GmbH, Goettingen, Germany) using 100×, 200×, and 400× magnification. The captured images were analyzed in terms of abundance and diameters of HFs, location of hair bulbs, and shape of hair shafts [14,29,59,69,70].

##### Statistical Analysis

GraphPad Prism version 7.04 for Windows (GraphPad Software, La Jolla, CA, USA) was used to conduct the statistical analysis. One-way ANOVA (analysis of variance) was used for multiple comparisons, followed by the Tukey–Kramer post hoc test [161]. *p* values ≤ 0.05 were considered significantly different. All parameters were tested at least in triplicate.

## 5. Conclusions

In this study, we provide evidence for the claimed hypothesis that the topical administration of agonists of drug efflux receptors encapsulated in chitosan nanoparticles is efficacious in preventing chemotherapy-induced alopecia. Moreover, they represent a more reliable strategy than using immune modulators such as tacrolimus or drug chelators such as N-acetylcysteine. We suggest further investigation of both phenobarbital (an agonist of CAR receptor that regulates the ABCB1 transporter) and pioglitazone (an agonist of PPAR γ) encapsulated in chitosan nanoparticles on human scalp cell lines, probably at a higher dose and for a prolonged dosage regimen.

## Figures and Tables

**Table 1:** A comparative summary of the efficacy of drug-loaded chitosan nanoparticles in the management of cyclophosphamide-induced alopecia in a mouse model.

Group	CIA ^1^ Induction	Treatment (Topical Application from Day 7 to Day 14 p.d. ^2^)	Primary Recovery (Day)	Secondary Recovery (Day)	Average HGI on Day 27	Hair Thickness on Day 27	**Recovery Pathway**	**Additional Notes**
Negative control	None	None	14	None	300 ± 0.00	26.25 ± 2.86	Normal anagen	Normal hair cycle timeline
Positive control	120 mg/kg cyclophosphamide by I.P. ^9^ injection on day 9 post-depilation	None	None	19	50 ± 0.00	18.89 ± 3.40	Dystrophic catagen	Secondary recovery still showed defective HFs ^10^ and shafts
PHB ^3^		F1	14	27	250 ± 50.99	33.62 ± 7.54	Dystrophic anagen	Most comparable to negative control
PGZ ^4^		F2		35	115 ± 33.17	17.72 ± 3.50		Best hair quality in primary recovery
RIF ^5^		F3		Not observed	97.5 ± 42.72	17.15 ± 2.62		Slowest recovery; white hair with poor morphology
NAC ^6^		F4		27	150 ± 86.79	16.96 ± 2.67		The second most effective
TAC ^7^		F5		Not observed	140 ± 77.78	20.18 ± 3.22		Effective primary recovery but delayed secondary recovery
Blank CS NPs ^8^		Blank CS NPs ^8^		Not observed	57.5 ± 66.52	26.25 ± 2.86		Minimal effect; regenerative role of chitosan only

^1^ Chemotherapy-induced alopecia; ^2^ post-depilation; ^3^ phenobarbital; ^4^ pioglitazone; ^5^ rifampicin; ^6^ N-acetyl cysteine; ^7^ tacrolimus; ^8^ chitosan nanoparticles; ^9^ intraperitoneal; ^10^ hair follicles.

**Table 2:** Compositions of the prepared drug-loaded chitosan nanoparticles.

	Drug	Conc. * (% *w*/*v*)	Polymer **	Crosslinker	Co-Solvent
F1	Phenobarbital	0.023	0.1% Chitosan in 1% acetic acid aqueous solution	0.05% Tripolyphosphate sodium	Propylene glycol
F2	Pioglitazone	0.020			
F3	Rifampicin	0.040			
F4	N-acetylcysteine	0.300			
F5	Tacrolimus	0.030			

* Each drug was added at an amount corresponding to its reported effective dose. ** The polymer/crosslinker/co-solvent ratio was kept constant at 5:2:3 in all formulations.

**Table 3:** The chromatographic conditions used to determine the entrapment efficiency of each formulation.

Formula (Drug)	Stationary Phase	Mobile Phase	Flow Rate (mL/min)	Oven Temperature	Photodiode-Array Detector Wavelength (nm)	Retention Time (min)	Reference
F1 (Phenobarbital)	Hypersil Gold column with dimensions 100 × 4.6, particle size 3 µm	Isocratic 100% acetonitrile	1.6	40	214	0.7	[137,141]
F2 (Pioglitazone)		Isocratic 100% methanol	1	25	225	1.227	[142,143,145]
F3 (Rifampicin)			1.3		235	0.7	[138,144,146]
F4 (N-acetyl cysteine)		Isocratic 95% acidic phosphate buffer, 5% acetonitrile	1	40	214	1.3	[139,147]
F5 (Tacrolimus)		Isocratic 100% acetonitrile	1.6	40	214	0.7	[137,141]

## Data Availability

Data is contained in the paper.

## Abbreviations

The following abbreviations are used in this manuscript:

ABC	ATP-binding cassette
ARA-c	Cytarabine
CAR	Constitutive androstane receptor
CIA	Chemotherapy-induced alopecia
CS	Chitosan
CS NPs	Chitosan nanoparticles
CYP	Cyclophosphamide
DL%	Drug loading percentage
EE%	Entrapment efficiency percentage
FT-IR	Fourier-transform infrared
HF	Hair follicle
NAC	N-acetyl cysteine
PGZ	Pioglitazone
PHB	Phenobarbital
PPARγ	Peroxisome proliferator-activated receptor gamma
PXR	Pregnane X receptor
PS	Particle size
RIF	Rifampicin
S.C.	Subcutaneous
TAC	Tacrolimus
TEM	Transmission electron microscope
ZP	Zeta potential

## Appendix A

**Table A1:** Construction of calibration curves using ultra-performance liquid chromatography for each of the used drugs: phenobarbital, pioglitazone, rifampicin, N-acetylcysteine, and tacrolimus.

Drug	Chromatogram	Calibration Curve
Phenobarbital (PHB)		
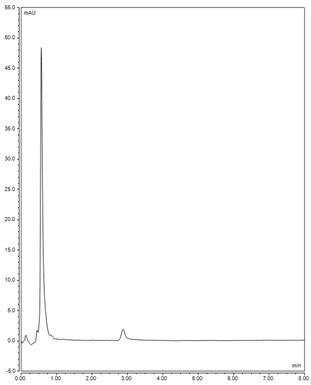
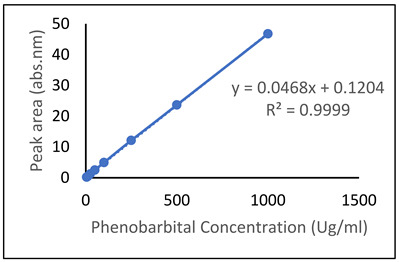
Pioglitazone (PGZ)		
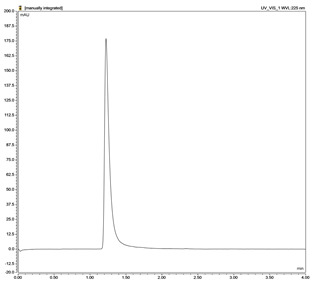
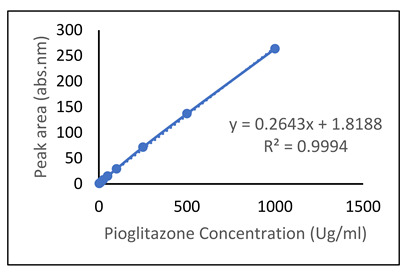
Rifampicin (RIF)		
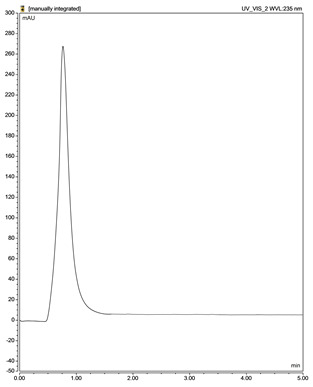
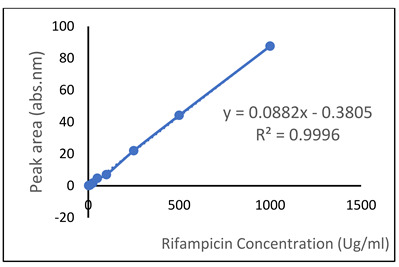
N-acetylcysteine (NAC)		
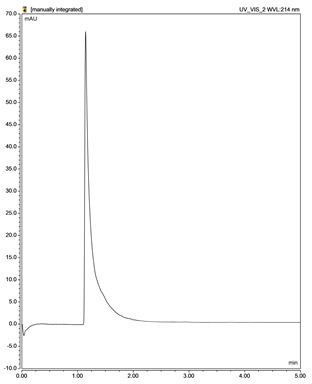
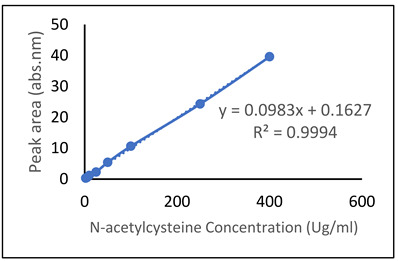
Tacrolimus (TAC)		
